# The Role of Heat Shock Protein 27 in Carcinogenesis and Treatment of Colorectal Cancer

**DOI:** 10.2174/1381612828666220427140640

**Published:** 2022-10-19

**Authors:** Fereshteh Asgharzadeh, Reyhaneh Moradi-Marjaneh, Mahdi Moradi Marjaneh

**Affiliations:** 1 Department of Physiology, School of Medicine, Mashhad University of Medical Sciences, Mashhad, Iran;; 2 Department of Physiology, School of Paramedical Sciences, Torbat Heydariyeh University of Medical Sciences, Torbat Heydariyeh, Iran;; 3 Department of Infectious Disease, Faculty of Medicine, Imperial College London, London, United Kingdom

**Keywords:** Carcinogenesis, colorectal cancer, heat shock protein 27, anticancer agents, chemotherapeutic agents, biomarker

## Abstract

The incidence of colorectal cancer (CRC) has significantly increased in recent decades, which has made this disease an important global health issue. Despite many efforts, there is no useful prognostic or diagnostic biomarker for CRC. Heat shock protein 27 (Hsp27) is one of the most studied members of the Hsp family. It has attracted particular attention in CRC pathogenesis since it is involved in fundamental cell functions for cell survival. Evidence shows that Hsp27 plays important role in CRC progression and metastasis. Hsp27 overexpression has been observed in CRC and is suggested to be associated with CRC’s poor prognosis. In the present review, we focus on the current knowledge of the role of Hsp27 in CRC carcinogenesis and the underlying mechanisms. In addition, we discuss the value of targeting Hsp27 in CRC treatment.

## 1 INTRODUCTION

Colorectal cancer (CRC) is the third most prevalent life-threatening cancer worldwide, and its diagnosis and treatment have become challenging. Surgery followed by chemotherapy may serve as the major approach for CRC treatment. However, these conventional therapeutic methods are unable to eradicate all cancer cells and have limitations due to toxic side effects. Thus, the identification of novel anticancer agents with less toxicity will improve the management of CRC. In the past decade, researchers have focused on identifying molecular mechanisms of CRC to reveal efficient prognostic, predictive, and therapeutic biomarkers [1, 2].

The heat shock protein 27 (Hsp27), also known as HspB1, is an understudied family of molecular chaperone proteins. This family is implicated in many different cellular functions such as refolding of unfolded proteins, regulation of cytoskeleton dynamics, and cell cycle regulation through direct interaction with several cellular proteins. There is growing evidence showing the involvement of the Hsp27 family in various types of cancer. Hsp27 overexpression can lead to the promotion of several aspects of tumorigenesis, such as elevation of cytoprotection, inhibition of apoptosis and cell death, resistance to chemotherapeutic agents, and metastasis. An increased expression of Hsp27 is closely associated with worse clinical outcomes in patients with CRC. Hsp27 overexpression is also shown to be related with resistance to chemotherapy drugs in CRC cells. Nevertheless, mechanisms underlying the role of Hsp27 overexpression in CRC remain poorly understood to date. Studies on the role of Hsp27 upregulation in CRC will improve our understanding of CRC pathogenesis [3-5].

In this review, we discuss a number of mechanisms through which Hsp27 may exert metastatic and invasive functions. Understanding these mechanisms may provide a new direction for the development of therapeutic strategies for CRC. We also summarize the recent knowledge about the therapeutic potential of targeting Hsp27 in CRC treatment.

## 2 CHARACTERIZATION OF HSP27

Hsp27 belongs to the small molecular weight heat shock protein (Hsp) family (sHsp, 15 to 30 kDa). This family is characterized by the inclusion in their C-terminal domains of a highly conserved sequence of 80-100 amino acids, “the α-crystalline domain”, flanked by less conserved (except a few stretches) N-terminal domains and a C-terminal extension [6]. Hsp27 is an ATP-independent chaperone encoded by the HspB1 gene. It is present in both the nucleus and cytoplasm as a multimeric complex. In normal cells, Hsp27 is expressed at low levels but is overexpressed when cells tolerate stress conditions, and plays an important role in controlling the balance between cell death and survival [7].

**Fig. (1):**
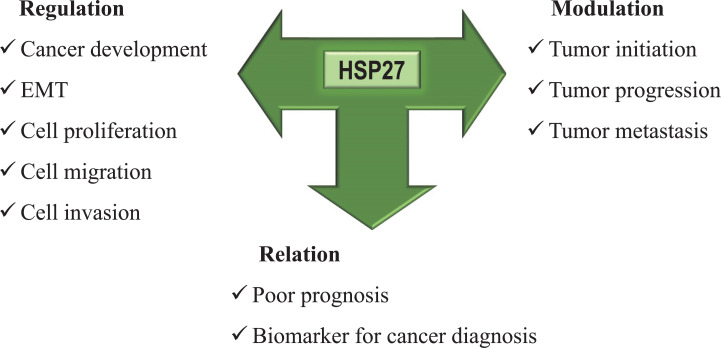
Summary of some of the major functions of Hsp27.

Hsp27 is involved in various processes; proper refolding of unfolded and damaged proteins is its primary function in response to various cellular stress conditions such as heat shock and chemical stress [5]. When cells are enduring oxidative stress conditions, Hsp27 acts as an antioxidant and reduces reactive oxygen through increasing intracellular glutathione and E-cadherin and decreasing intracellular iron [8]. The protein functions as an antiapoptotic agent through direct interactions with proteins involved in extrinsic and intrinsic apoptosis pathways. Hsp27 suppresses Bax or Daxx and mostly attenuates Caspase-3 activity [9]. It also inhibits apoptosis by promoting NF-κB activity by increasing the degradation of its main inhibitor, I-κBα [10-12]. In addition, Hsp27 performs cytoprotection against programmed cell death. It is also capable of regulating actin cytoskeletal dynamics through actin polymerization and acts as an actin capping protein (Fig. **1**) [3, 5]. The cytoprotection functions of Hsp27 against apoptosis and cell death suggest that Hsp27 is partially responsible for cell survival. These features indicate its fundamental role in cancer pathophysiology [4]. Hsp27 is upregulated in many types of cancer and is associated with progression, metastasis, drug resistance, and poor prognosis [4].

**Fig. (2):**
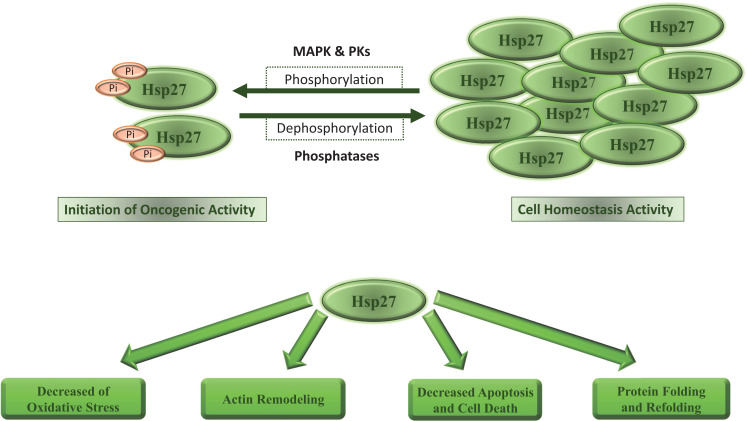
Role of HSP27 in cancer development.

The dynamics between the two forms of Hsp27 (phosphorylated and unphosphorylated) influence its ability to confer stress protection. Hsp27 exists as large unphosphorylated oligomers within cells. It participates in chaperoning function and maintaining the homeostasis in this state [4]. Recent studies show that Hsp27 has the most chaperone activity and binding to other client proteins in the dimer state [13, 14]. However, it is phosphorylated in response to various stressors, which results in dissociation of the oligomers complex, and promotes the formation of small oligomers [4]. Hsp27 phosphorylation is performed at specific serine residues by multiple kinase pathways, including activated mitogen-activated protein kinase (MAPK), protein kinase B (PKB), protein kinase C (PKC), protein kinase D (PKD), and protein kinase G (PKG). The phosphorylation process is regulated by several factors including interleukin-1β (IL-1 β), tumor necrosis factor-α (TNF-α), transforming growth factor-beta (TGF-β), and mitogens, such as insulin-like growth factor-1 (IGF-1) and steroid hormones [15, 16]. It is reported that immediate and transient phosphorylation of Hsp27 is underlying its pro-oncogenic and chemoresistance functions (Fig. **2**) [15, 17].

## 3 HSP27 AND CRC

Increased expression of Hsp27 in CRC cells can lead to more malignant tumors. This was investigated by Garrido *et al*. who used two rat CRC cell lines, PROb, and REGb. Unlike REGb, PROb cells expressed a high level of Hsp27. An injection of PROb cells into syngeneic rats caused progressive tumors. However, REGb cells formed tumors that regressed within a few weeks. As another piece of evidence of the Hsp27 tumor potential, they transfected REG cells with Hsp27 cDNA and showed Hsp27 overexpression inhibited apoptosis and caused large tumors [18].

Hsp27 expression in CRC tissues and surrounding normal mucosa varies [19]. Yu *et al*. showed that Hsp27 upregulation is common in human colon cancer tissues [20]. From 182 paraffin-embedded CRC samples, 145 (79.7%) were positive for Hsp27 staining in tumor cells. Another statistical study of the relationship between Hsp27 expression and clinicopathological characteristics of CRC patients revealed a strong association between Hsp27 expression and TNM staging of malignant tumors. These findings suggest Hsp27 as a biomarker for CRC patients with poorer clinical outcomes. Yu *et al*. also showed that patients with higher Hsp27 expression had a poorer prognosis compared with those with lower Hsp27 expression. Moreover, after regressing the effect of TNM stage and adjuvant chemotherapy, they suggested Hsp27 overexpression as an independent prognostic indicator for overall survival [20]. Similar results were reported by Wang *et al*. who examined Hsp27 expression by immunohistochemistry in 175 primary CRC tumors and their corresponding normal mucosa samples and found a relationship between Hsp27 expression and adverse outcomes in CRC patients [21].

The role of Hsp27 in developing resistance to cell death and drug treatment in CRC has been a focus of many studies. CRC cells with high Hsp27 expression show increased apoptotic resistance and tumorigenicity [22]. Furthermore, Hsp27 is involved in irinotecan and 5-FU resistance in CRC [23, 24]. Overexpression and suppression of Hsp27 have been shown to increase and decrease resistance to 5-FU, respectively, in colon cancer cells using small interfering RNA (siRNA) and small hairpin RNA (shRNA) [24, 25].

Growing evidence shows that Hsp27 has a significant role in CRC metastasis [26-28]. A microarray study of colorectal cancer including 81 CRC paired samples (carcinomatous and non-neoplastic human colorectal tissues) analyzed Hsp27 protein levels. It showed an increased staining density of Hsp27 in carcinomatous relative with non-neoplastic tissues. In addition, high Hsp27 levels were associated with poor prognosis as shown by a Kaplan-Meier analysis suggesting Hsp27 as a major mediator in CRC metastasis and invasion. Accordingly, suppression of Hsp27 by shRNA-mediated genes reduced cell proliferation and invasion. Based on the data, it was hypothesized that Hsp27 could be a prognostic marker with a pro-metastatic feature in CRC [28].

Inconsistent with these reports, in a study of paired human colon adenocarcinoma cell lines, SW480 (primary/pre-metastatic tumor) and SW620 (lymph node metastasis), Zhao *et al*. showed that, unlike SW620 cells, SW480 cells released Hsp27, indicating Hsp27 expression may be inversely related with the metastatic activity of CRC cells. In addition, they reported that Hsp27 expression was not correlated with clinicopathological factors including age, gender, histological grade, lymphatic metastasis, and clinical stage. However, its overexpression had a negative relationship with lymphatic metastasis [27].

Hsp27 can therefore be a novel therapeutic target for CRC treatment and controlling metastasis. However, the specific mechanism underlying the role of Hsp27 in CRC progression and metastasis has not been determined yet. Here are some possible mechanisms that have been reported in the literature.

## 4 HSP27 AND EPITHELIAL-MESENCHYMAL TRANSITION (EMT)

The epithelial-mesenchymal transition (EMT) is a cell remodeling process by which epithelial cells lose their polarized organization and cell-cell adhesion, and gain migratory and invasive capabilities [29]. EMT has an important role in CRC invasion and metastasis [30]. Evidence suggests that Hsp27 is probably involved in regulating EMT and thus the invasion and metastasis of CRC. Han *et al*. investigated the role of Hsp27 in the invasion and proliferation of CRC cells. They found that Hsp27 knockdown by lentivirus-mediated shRNA impaired the proliferation and invasion of CRC cells *in vitro* and *in vivo*, while its overexpression augmented the proliferation and invasion of CRC cells *in vitro*. A significant finding of the Han study was that Hsp27 could drive EMT independently in CRC cells [28]. Hsp27 silencing increased the expression of epithelial markers and decreased the expression of mesenchymal markers. Consistently, Hsp27 overexpression in HT29 cells increased the expression of mesenchymal markers [28]. Collectively, these results showed that Hsp27 overexpression induced aggressiveness and metastatic features in CRC cells by driving EMT [28]. The role of Hsp27 in EMT was also demonstrated by Cordonnier *et al*. in prostate cancer. They showed that Hsp27 was required for epidermal growth factor (EGF) signaling-induced cell migration, invasion, and matrix metallopeptidases (MMPs) activity. In addition, they showed that Hsp27 was involved in the downregulation of E-cadherin with simultaneous stimulation of the expression of EMT markers including fibronectin, vimentin, and slug. Hsp27 is also involved in the activation of AKT, phosphorylation of GSK3β, and β-catenin nuclear entry. Furthermore, silencing Hsp27 reduced EGF-dependent phosphorylation of β-catenin on tyrosine 142 and 654, increased β-catenin ubiquitination and degradation, and inhibited β-catenin nuclear translocation [31]. From these results, it can be concluded that Hsp27 is involved in the regulation of Wnt signaling. Wnt signaling is a crucial pathway that is hyperactive in CRC [32]. Furthermore, they suggest that Hsp27 regulates EMT through modulation of the β-catenin/Slug signaling pathway [31]. Similar results on the role of Hsp27 in EMT were reported by Wei *et al*. in breast cancer stem cells. They showed that the knockdown of Hsp27 suppressed EMT signatures, including attenuating the expression of snail and vimentin and augmenting the expression of E-cadherin, which was concomitant with a reduction in the features of breast cancer stem cells. In addition, they found that Hsp27 could modulate the nuclear entry and activity of NF-κB in breast cancer stem cells by increasing the expression of IκBα [33]. In addition, the expression of Hsp27 in CRC was strongly related with the co-presence of wild-type *KRAS* and activated phosphoinositide 3-kinases (PI3Ks)/AKT, indicating a possible role of Hsp27 in overcoming PI3K/AKT oncogene-induced senescence in CRC tumors [34].

## 5 HSP27 AND CALCIUM CHANNEL SIGNALING

Calcium channels are essential for cell metabolism regulation [35]. Various studies indicate the important role of Ca^2+^ and Ca^2+^ ion channels in augmenting proliferation, invasion, motility, and metastasis in various types of cancers including CRC [36]. Stromal Interaction Molecule 1 (STIM1) and Calcium Release-Activated Calcium Modulator 1 (ORAI1) are involved in the management of the mechanism of Ca^2+^ signaling, the store-operated calcium entry (SOCE) [37, 38]. STIM1 functions as a Ca^2+^ sensor on the endoplasmic reticulum (ER) which regulates the Ca^2+^ input process in response to external stimuli [39]. The reduction of calcium level in the ER is sensed by STIM1, thus STIM1 clusters form “puncta” and supplant close to the plasma membrane of the cell, where it activates ORAI1, a calcium selective ion channel [40, 41]. Previous research highlighted the crucial role of STIM1 in tumorigenesis. Targeting STIM1 has been shown to inhibit metastasis of hepatocellular carcinoma, melanoma, breast cancer, and CRC tumors [42-46].

Huang *et al*. showed that Hsp27 was a chaperone that may interact with STIM1 but not ORAI1, and stabilized the SOCE's STIM1 and targeting Hsp27 inhibited G2/M transformation which is associated with reduced cancer proliferation, invasion, and metastasis [47]. They showed that Hsp27 knockdown in CRC cells decreased cell growth through arresting the G2/M transformation. This G2/M arrest was then attributed to changes in molecules involved in the cell cycle. The cell cycle is a highly controlled process [48]. The G2 checkpoint is essential in DNA repair during cancer, with several targets for the G2/M checkpoint being under study [49, 50]. According to Huang *et al*. results, Hsp27 modified levels can affect cell cycle regulation and, consequently, the survival of cancer cells, suggesting targeting Hsp27 may improve current CRC treatments [47].

## 6 HSP27 AND ANGIOGENESIS

Angiogenesis enhancement is another role of Hsp27. To explore the extracellular proangiogenic effect of Hsp27 in CRC and breast cancer cells, Thuringer *et al*. tested the recombinant human protein (rhHsp27) in human microvascular endothelial cells (HMECs) formed as monolayers or spheroid models. They showed that Hsp27 promoted angiogenesis through TLR3-dependent calcium entry and Nf-κB activation in human endothelial cells, leading to subsequent secretion of vascular endothelial growth factor (VEGF), which is the main regulator of cancer angiogenesis, and activation of VEGFR2 and interleukin-8 (IL-8), which are the proangiogenic factors in endothelial cells. It also contributes to cell migration and tubulogenesis [51]. Activation of TLR3 can also promote angiogenesis through hypoxia-inducible factor 1α (HIF1α) [52]. Metastatic CRC has been shown to induce systemic Hsp27 expression. Removing pulmonary metastases resulted in a significant reduction in total Hsp27 and hosphor-Hsp27 (a polymerized form of Hsp27) levels. Additionally, preoperative total Hsp27 and hosphor-Hsp27 levels were higher compared with matched healthy controls and declined after surgery.

## 7 HSP27 AND TUMOR MICROENVIRONMENT

Cancer-associated fibroblasts (CAFs; also called tumor-associated fibroblasts) are myofibroblasts and fibroblasts within the tumor microenvironment that promote carcinogenic features by initiating extracellular matrix remodeling or by secreting growth factors and cytokines [53]. CAFs have been reported to act as prognostic and predictive markers in several cancers including CRC. Activated CAFs express high levels of Hsp27, which is crucial for fibroblast adhesion, contractility, and motility [54, 55]. Schweiger *et al*. revealed for the first time that Hsp27 was overexpressed in the tumor stroma of CRC. Stromal α-SMA, which is established as a marker for fibroblast activation, and Hsp27 expression were strongly correlated with each other and related with worse clinical outcomes including recurrence-free survival and overall survival after pulmonary metastasectomy. Moreover, serum levels of phospho Hsp27, the polymerized form of Hsp27, and total Hsp27 decreased after metastasectomy to levels comparable with healthy controls, indicating the potential value of serum Hsp27 as a prognostic marker for metastatic CRC [56]. However, it was not correlated with systemic CRP or fibrinogen levels. Therefore, circulating Hsp27 is potentially affected by other factors including secretion and degradation. Further studies are required to more precisely explain the possible prognostic and predictive role of circulating Hsp27 in CRC patients [56].

## 8 TARGETING HSP27 IN CRC TREATMENT

Considering the role of Hsp27 protein in CRC pathogenesis, targeting this by inhibitor compounds can be a new window in CRC treatment. Unlike other ATP-binding Hsps, however, Hsp27 is an ATP-independent chaperone which makes it difficult to be targeted with small compounds [26]. Hsp27 inhibitors are categorized into small molecule inhibitors, antisense oligonucleotides (ASO), and peptide aptamers (Table **1**).

Several small molecules such as Hsp27 inhibitors are currently under evaluation, including quercetin, RP101, zerumbone, sw15, and J2 (Table **1**). Quercetin is a flavonoid compound that comes from nature, and has anticancer properties through suppressing heat shock transcriptional factor1 (HSF1)-dependent Hsps in various cancer cell lines [57-59]. Although no exact mechanism associated with Hsp27 has been yet identified, it has been suggested that quercetin might have a possible role as a regulator of Hsp27 protein stability by inhibiting cellular expression of casein kinase 2 (CK2) [60]. A clinical trial titled “sulindac and plant compounds in preventing colon cancer” (NCT00003365) has tried to evaluate the role of quercetin in the prevention of CRC. However, the results are not available yet at “clinicaltrials.gov.” Another herbal compound with an inhibitory effect on Hsp27 is 1,3,5-trihydroxy-13,13-dimethyl-2H-pyran xanthone (TDP) which is extracted from a Chinese medicinal herb, Garcinia oblongifolia. TDP- induced apoptosis by suppressing the Hsp27 expression that was specifically related with the mitochondrial death of the caspase-dependent pathway in hepatocellular carcinoma [61]. RP101, known as BVDU (bromovinyldeoxyuridine, brivudine), is an antiviral nucleoside that forms π-stacking binding to Phe29 and Phe33 of Hsp27 to inhibit its function. This connection weakens the binding of Hsp27 to Akt1, pro-caspase3, and cytochrome C, and promotes apoptosis [62, 63]. A phase II clinical trial to evaluate the efficacy and safety of RP101 in combination with gemcitabine in pancreatic cancer (NCT00550004) showed promising results in the prevention of chemoresistance and enhancement of chemosensitivity [62]. Heinrich *et al*. used a novel computational drug repositioning approach to exploit the similarities between a predicted Hsp27 binding site and a viral thymidine kinase (VTK). They generated lead inhibitors for Hsp27, of which six bound Hsp27 and downregulated its chaperone activity. They were from these groups of compounds: BVDU, thymine, deoxyuridine, guanine, and phenothiazine. In addition, all six agents inhibited drug resistance and chaperone activity better than BVDU (50-80 times). They also showed that the bromovinyl group of BVDU is not necessary for Hsp27 inhibition. Moreover, an Hsp27 inhibitory activity was shown for chlorpromazine (an antipsychotic agent) [63].

Altered cross-linking of Hsp27 proteins is a new strategy for inhibiting Hsp27 [64]. This was done by inserting an agent between the disulfide bond of Hsp27 and disrupting the normal Hsp27 dimerization which functionally inhibited Hsp27 [65]. This may improve drug production, which is currently limited by the absence of Hsp27 inhibitors. Some compounds including zerumbone (ZER), SW15, and J2 use the alteration of cross-linking to inhibit Hsp27-mediated resistance. Zerumbone, a phytochemical isolated from a natural compound, has been shown to produce cross-linking of Hsp27. Choi *et al.* placed ZER between the disulfide bond in the Hsp27 dimer and reduced the affinity between Hsp27 and apoptotic molecules [64]. SW15, a more potent Hsp27 cross-linker, is a synthetic xanthone compound [66]. Following ZER and SW15, J2 was suggested for altered cross-linking of Hsp27 proteins. J2 is a chromene compound and exhibited a better cross-linking activity of Hsp27 compared with zerumbone and SW15. It showed a potent synergism with Hsp90 inhibitors and conventional anticancer drugs [65]. In addition, anti-inflammatory and antioxidant properties of J2 have been reported and it has been suggested as a therapeutic agent for radiation-induced lung injury [67]. Further studies are needed before using small molecule Hsp27 inhibitors in the treatment of CRC patients.

The second category of Hsp27 inhibitors is antisense drugs. Antisense drugs have been used to describe single-stranded oligonucleotides (ss-ASos) designed to hybridize RNAs [68]. A second-generation phosphorothioate ASO targeting Hsp27 mRNA is apatorsen (OGX-427) with the chemistry of 2'-O-methoxy-ethyl Bases (2'-MOE). Apatorsen showed promising results in phase I and II clinical trials in prostate cancer (NCT00487786 and NCT01120470) [69, 70]. However, addition of apatorsen to the standard carboplatin/pemetrexed regimen in metastatic nonsquamous NSCLC patients did not improve the outcome [71]. The evaluation of the effectiveness of apatorsen in CRC requires experimental and clinical studies.

The third category of Hsp27 inhibitors is peptide aptamers, which are small amino acid sequences inserted into a scaffold protein. Peptide aptamers could bind to and disrupt the dimerization and oligomerization of Hsp27 and inhibit the antiapoptotic activity of Hsp27. Two peptides, PA11 and PA50, could act as negative regulators of Hsp27 functions [72, 73]. Although these compounds are not effective alone, experimental studies showed that they could increase radiosensitivity and cell death when used in combination with other anti-cancerous agents [74]. The preclinical success of peptide aptamers suggests that they have the potential of being a new avenue of cancer therapy.

Thiolutin is a naturally occurring bicyclic dithiole that exerts its anti-cancerous activity by inhibiting endothelial cell adhesion and angiogenesis through rapid induction of Hsp27 phosphorylation. Studies have shown that Hsp27 plays a role in both the assembly and disassembly of actin filaments. Phosphorylated Hsp27 permits actin polymerization, while non-phosphorylated Hsp27 inhibits actin polymerization. Thiolutin induces loss of actin stress fibers, increases cortical actin as cells retract, reduces cellular F-actin, enhances peripheral localization of phosphorylated Hsp27, ablates Hsp27 interaction with nestin, and collapse nestin filaments [75, 76].

## CONCLUSION

Hsp27 is a chaperone that interacts with many proteins and protects cells against many environmental and physical stressors. Deregulated expression of Hsp27 has been repeatedly observed during cancer progression. Hsp27 expression has been reported to be associated with poor survival in different cancer types including CRC, esophageal adenocarcinoma, and non-small cell lung carcinoma, and may be an independent prognostic factor [20, 77, 78]. CRC patients with high Hsp27 expression can be candidates for aggressive treatment. Although the detailed mechanisms of involvement of Hsp27 in CRC are still unclear, researchers report its role in the regulation of epithelial-mesenchymal transition (EMT), calcium channel signaling, angiogenesis, and tumor microenvironment.

The specific functions of the signaling pathways, proliferation, survival, and acquired resistance to chemotherapy in CRC cells indicate that Hsp27 could be a potential CRC therapeutic target with appropriate inhibitors. The search for the discovery of Hsp27 inhibitors led to the identification of a variety of Hsp27 inhibitors categorized into small molecule inhibitors such as quercetin, TDP, RP101, zerumbone, SW15, and J2; antisense oligonucleotides (ASO) including apatorsen and peptide aptamers such as thiolutin, PA11, and PA50 (Table **1**). Some of these compounds such as quercetin, RP101, and apatorsen are under clinical trials to evaluate their efficacy in cancer treatment. The others need more experimental studies before being used in the treatment of CRC.

## Figures and Tables

**Table 1:** The structure of heat shock protein 27 (Hsp27) inhibitors.

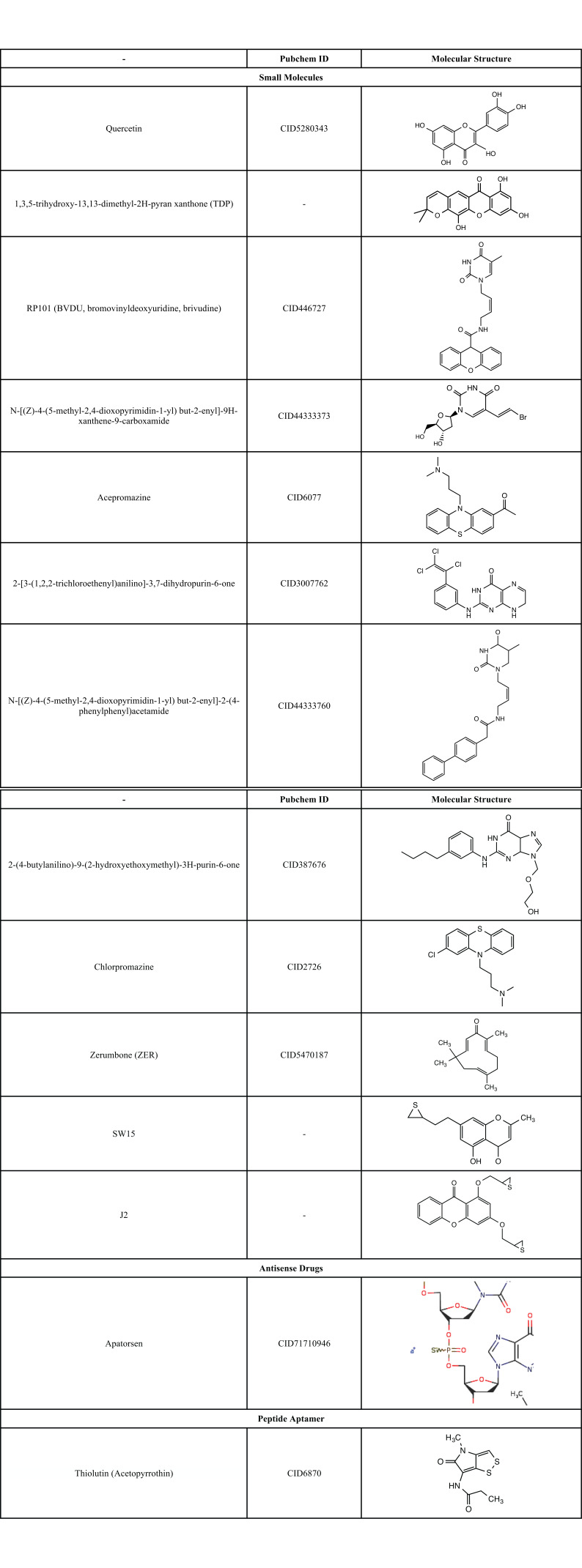

## LIST OF ABBREVIATIONS

ASO: Antisense Oligonucleotides
CK2: Casein Kinase 2
CRC: Colorectal Cancer
EGF: Epidermal Growth Factor
EMT: Epithelial-mesenchymal Transition
HIF1α: Hypoxia-inducible Factor 1α
Hsp27: Heat Shock Protein 27
IGF-1: Insulin-like Growth Factor-1
IL-1β: Interleukin-1β
MAPK: Mitogen-activated Protein Kinase
MMPs: Matrix Metallopeptidases
PKB: Protein Kinase B
PKC: Protein Kinase C
PKD: Protein Kinase D
PKG: Protein Kinase G
shRNA: Small Hairpin RNA
siRNA: Small Interfering RNA
STIM1: Stromal Interaction Molecule 1
TGF-β: Transforming Growth Factor-beta
TNF-α: Tumor Necrosis Factor-α
VEGF: Vascular Endothelial Growth Factor
VTK: Viral Thymidine Kinase
